# Regulation of Autophagy through TORC1 and mTORC1

**DOI:** 10.3390/biom7030052

**Published:** 2017-07-07

**Authors:** Takeshi Noda

**Affiliations:** Center for Frontier Oral Science, Graduate School of Dentistry, Osaka University, Suita, Osaka 565-0871, Japan; takenoda@dent.osaka-u.ac.jp

**Keywords:** autophagy, TORC1, Atg13, Atg1, Atg9, Atg8, PAS, Gln3, TFEB

## Abstract

Autophagy is an intracellular protein-degradation process that is conserved across eukaryotes including yeast and humans. Under nutrient starvation conditions, intracellular proteins are transported to lysosomes and vacuoles via membranous structures known as autophagosomes, and are degraded. The various steps of autophagy are regulated by the target of rapamycin complex 1 (TORC1/mTORC1). In this review, a history of this regulation and recent advances in such regulation both in yeast and mammals will be discussed. Recently, the mechanism of autophagy initiation in yeast has been deduced. The autophagy-related gene 13 (Atg13) and the unc-51 like autophagy activating kinase 1 (Ulk1) are the most crucial substrates of TORC1 in autophagy, and by its dephosphorylation, autophagosome formation is initiated. Phosphorylation/dephosphorylation of Atg13 is regulated spatially inside the cell. Another TORC1-dependent regulation lies in the expression of autophagy genes and vacuolar/lysosomal hydrolases. Several transcriptional and post-transcriptional regulations are controlled by TORC1, which affects autophagy activity in yeast and mammals.

## 1. Introduction

In 2016, the Nobel Prize for physiology and medicine was awarded to Dr. Yoshinori Ohsumi for the discovery of the mechanism of autophagy. This award was interpreted as substantiating the concept that life is maintained in a continuous flux between protein synthesis and degradation. This concept was originally established by Rudolf Schoenheimer in the 1940s [1]. Autophagy, the self-degradation system, was first discovered in mammalian cells in 1960s [2]; however, limited research had been conducted, until yeast research on autophagy started. The Ohsumi lab, along with the Klionsky and Thumm labs, identified a group of autophagy-related genes (*Atg*), which encoded proteins that were mostly involved in autophagy membrane biogenesis in yeast [3,4,5]. These *Atg* genes are well conserved in eukaryotes; this enabled further research on autophagy in mammalian systems. This research revealed that autophagy is linked to a diverse pathophysiology, highlighting the value of Schoenheimer’s pioneering dogma, driving the question of how the catabolic process of autophagy and the anabolic process of protein synthesis are coordinated. The short answer is that target of rapamycin complex 1 (TORC1) coordinates them. The long answer will be described in detail in the series of reviews in this volume; in this particular review, I will describe how TORC1 regulates autophagy.

The link between rapamycin, the specific inhibitor of TORC1, and autophagy was first described in rat hepatocytes by Alfred Meijer’s group [6]. Rapamycin treatment abrogated the suppressive effect of amino acids toward starvation-induced autophagy. As it also prevented protein synthesis through ribosomal protein S6 (rpS6) dephosphorylation, the relationship between autophagy and TOR was still unclear. Direct involvement of TOR in autophagy regulation was shown in yeast [7]. Even under nutrient-rich conditions where autophagy is not induced, rapamycin treatment or TORC1 inactivation alone was sufficient for autophagy induction. Thus, the catabolic process of autophagy and the anabolic process of protein synthesis were controlled through a single molecular switch, which is a reasonable mechanism for avoiding a futile cycle between protein synthesis and degradation, although some exceptional situations are also reported such as autophagy and protein synthesis simultaneously induced in some tumor cells [8].

## 2. Atg13–Atg1/ULK1

**Figure 1:**
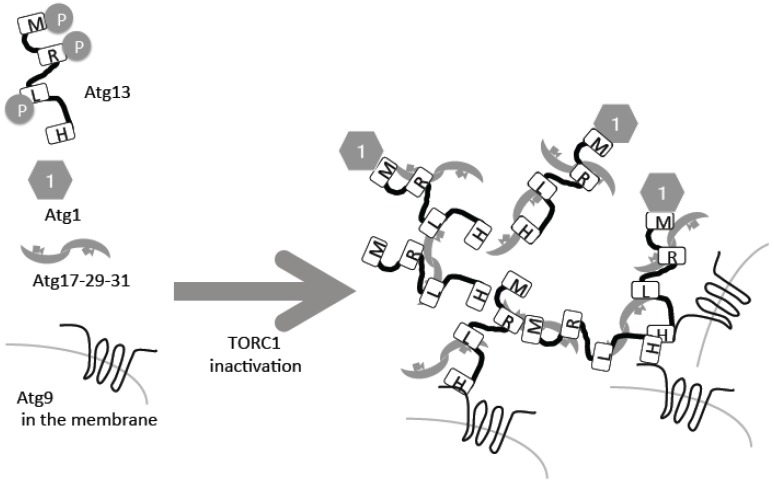
Autophagy related gene 13 (Atg13) dephosphorylation leads to preautophagosomal structure (PAS) formation. Atg13 is phosphorylated by target of rapamycin complex 1 (TORC1), and inactivation of TORC1 leads to its dephosphorylation, resulting in PAS formation. For details, please see the text. H: HORMA domain; R: Atg13-17BR; L: Atg13-17BL; M: MIM domain.

The direct substrate of TORC1 in yeast is Atg13, which is hyper-phosphorylated under nutrient-rich conditions (Figure 1) [9,10,11]. Following inactivation of TORC1 under starvation conditions, it is rapidly dephosphorylated (Figure 1). The phosphatase responsible for this dephosphorylation is still unknown, but two forms of protein phosphatase 2A (PP2A), PP2A–Cdc55 and PP2A–Rts1, have been implicated [12]. Atg13 is phosphorylated also by another kinase, protein kinase A, which negatively regulates autophagy too [13]. Atg13 is a serine-rich protein that contains several phosphorylation sites and other critical regions [14]. Two Atg13 regions, Atg13-17BR and Atg13-17LR, bind to another autophagy protein, Atg17, which forms a complex with Atg29 and Atg31 [14,15]. This trimetric complex forms homo dimers (Figure 1) [16,17]. Phosphorylated residues in different regions of Atg13 bind to Atg17 weakly, but upon dephosphorylation, the interactions are strengthened [14,15]. When the Atg13–Atg17 interactions are strengthened, intermolecular bridges of different Atg17–Atg23–Atg31 hexamers facilitates formation of super molecular complexes of Atg13 and Atg17–Atg29–Atg31, consisting of around 50 complexes each (Figure 1) [15].

Atg13 has another region called the MIM domain, which binds the protein kinase Atg1, the first autophagy-related gene to be cloned [14,18]. Phosphorylation in the MIM domain weakens this interaction; therefore, as in the case of Atg17, the Atg13–Atg1 interaction is facilitated by starvation in a TORC1-dependent regulation manner (Figure 1) [10,14]. Atg1 kinase activity is essential for autophagy [18]. Atg1 kinase activity is minimal under nutrient-rich conditions, thus suppressing autophagy. More precisely, minimal Atg1 kinase activity is required for a selective autophagy called the cytoplasm-to-vacuole (Cvt) pathway, which also occurs in nutrient-rich conditions [10]. Under nutrient starvation conditions, Atg1 kinase activity is upregulated and autophagy is induced [10]. This regulation is conducted by self-association of different Atg1 molecules and auto-phosphorylation [19]. Therefore, it is proposed that incorporation of Atg1 molecules into super Atg13–Atg17–Atg29–Atg31 complexes enables self-association of intermolecular Atg1, resulting in up-regulation of Atg1 protein kinase activity [15]. This super complex forms the pre-autophagosomal structure/phagophore assembly site (PAS), which serves as a scaffold for autophagosome formation and facilitates recruitment of the other Atg molecules that will be further described below [20,21].

In mammalian cells, rapamycin treatment alone was shown to be sufficient for autophagy induction [22]. Soon after the discovery of autophagy machinery in yeast, the hunt for the mammalian homologues was initiated. Early on, Ulk1, the Atg1 protein kinase homologue, was identified [23]. It was first identified as a homologue of unc-51 kinase in *Caenorhabditis elegans*, implicated in an uncoordinated locomotion phenotype independent of autophagy [23]. Subsequently, several groups have reported Atg13 homologues [24,25,26], but none have reported on Atg17. However, focal adhesion kinase family interacting protein of 200 kD (FIP200) has been reported to serve similar roles as Atg17 [27]. Together with another subunit—Atg101—Atg13 and FIP200 form a complex with Ulk1 [28,29]. While the Atg1 complex in yeast mostly disassembles in nutrient-rich conditions, the Ulk1 complex in mammalian cells does not [24]. Under nutrient-rich conditions, the Ulk1 complex directly associates with mTORC1 via its subunit Raptor, resulting in Ulk1 and mAtg13 phosphorylation by mTORC1 [24,25]. Under starvation conditions, Ulk1 is dephosphorylated and dissociates from mTORC1, resulting in auto-phosphorylation and upregulation of kinase activity. Ulk1 then phosphorylates Atg13 and FIP200 [24,25,26]. Possibly coupling with some of these phosphorylation statuses, the ULK1 complex gathers to the specific endoplasmic reticulum (ER) region engaged in autophagosome formation [30], which may correspond with PAS formation in yeast.

## 3. Atg9

In addition to auto-phosphorylation, an important substrate of the Atg1 protein kinase, which is regulated by TORC1, is Atg9 [31]. Atg9 is a transmembrane protein with six membrane-spanning domains [32], and is found on transport vesicles between Golgi and endosomes. In response to autophagy, a portion of Atg9 is recruited to the PAS [33,34], where it is integrated into the outer autophagosomal membrane. After autophagy is complete, Atg9 returns to the cytosol [35]. It is still under debate how Atg9 reaches the PAS; one possibility is that Atg9-residing transport vesicles are gathered directly to the PAS [34,35]. At the amino-terminal of Atg13, the HORMA domain directly binds to the amino-terminal cytosolic stretch of Atg9, but this process is not regulated by Atg13 phosphorylation [36]. When Atg9 reaches the PAS, it is bound by Atg13 (Figure 1). In addition to Atg13, Atg17 is also suggested to be directly associated with Atg9 [37]. Therefore, Atg9 may be retained at the PAS in a multi-modal manner. Mutating the Atg1-dependent phosphorylation site prevents autophagy, possibly through a defect in its interaction with Atg18, which is a phosphatidylinositol 3-phosphate (PI3P) binding protein [31]. Importantly, even without the Atg1 complex, Atg9 can reach the PAS, meaning that the recruitment process itself is not regulated by Atg1 and Atg13 [33]. On the contrary, without the Atg1 complex, Atg9 cannot return to the cytosolic pools [33]. A similar relationship between Atg9 and Ulk1 is also observed in mammalian cells [38]. The Atg13 HORMA domain-dependent binding to PAS and subsequent Atg1-mediated phosphorylation is crucial for autophagosome formation, and without it, Atg9 cannot return to the cytosolic pools. As a result of this initiation event, formation of the autophagosomal membrane begins, resulting in recruitment of Atg18–Atg2, PI3P kinases, Atg12–Atg5–Atg16 and Atg8. The involvement of TORC1-dependent processes in these later stages is scarcely known (except for the expression control of Atg8 described below), and will be described in detail elsewhere [39,40]. 

## 4. Change in TORC1 Localization

**Figure 2:**
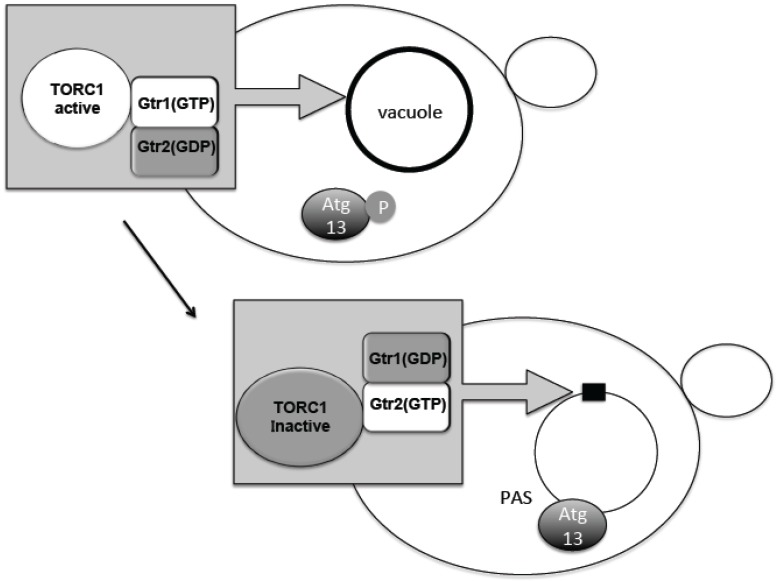
Dynamic relocation of TORC1 is associated with its activity. When Gtr1 takes guanosine 5’-triphosphate (GTP) form, TORC1 is active and spread over the vacuole. When Gtr2 takes GTP form, TORC1 is inactive and concentrated on a punctuated structure on the vacuole. When TORC1 is active Atg (autophagy related gene) 13 is phosphorylated, and does not gather to preautophagosomal structure (PAS). When TORC1 is inactive, Atg13 is dephosphorylated and gathers to PAS. GDP; guanosine 5’-diphosphate.

The TORC1–Atg13 relationship constitutes the main axis of autophagy regulation. It was therefore important to know where regulation occurs with respect to subcellular localization. Phosphorylated Atg13 proteins under nutrient-rich conditions showed dispersed distribution throughout the cytoplasm. Upon TORC1 suppression by starvation, dephosphorylated Atg13 is supposed to move to the perivacuolar region at the PAS (Figure 2) [15]. TORC1 is concentrated on the vacuolar membrane, and PAS is always attached to the vacuolar membrane (Figure 2) [20,41]. Active TORC1 on the vacuolar membrane will prevent dephosphorylation of Atg13 and association to the PAS. Recently, we have uncovered an interesting localization shift of TORC1. TORC1 is localized to the vacuole via binding to the scaffold protein complex EGO1-2-3 and Gtr1-2 [42,43,44]. Gtr1 and Gtr2 form a heterodimeric guanosine 5’-triphosphate (GTP)ase and differentially bind either GTP or guanosine 5’-diphosphate (GDP), i.e., when Gtr1 binds GTP, Gtr2 binds GDP and vice versa (Figure 2) [45]. These binding states are correlated with the in vivo activation states of TORC1. When Gtr1 binds GTP, TORC1 is active and when Gtr2 binds GTP, TORC1 becomes inactive (Figure 2) [46]. Interestingly, when Gtr1 binds GTP, TORC1 becomes localized over the vacuolar membrane [43,47]. However, when Gtr2 binds GTP, the vacuolar localization becomes dispersed and forms mostly single, punctate localization along the vacuolar membrane (Figure 2) [43,47]. This Gtr2-dependent punctate localization is mediated through the direct binding of Gtr2 and the TORC1 subunit Kog1 [43,47]. Consistently, the localization is correlated with the nutrient condition, and by nutrient starvation, the punctate localization increases (Figure 2) [43,47]. Although a detailed molecular mechanism of TORC1 activity regulation is still unclear, it is conceivable that during starvation, TORC1 is sequestered to the puncta, which prevents Atg13 access to the PAS. When nitrogen is added to the starved yeast cells, Atg13 is phosphorylated and, at the same time, TORC1 localization becomes punctate along the vacuolar membrane localization [10,48]. Thus, it is proposed that at least part of autophagy regulation by TORC1 is due to a change in subcellular localization. By contrast, mTORC1 localization is dynamically changed between lysosomal membrane surface and the cytosolic pool, depending on amino acid availability [49]. This localization shift is regulated by Rag GTPases, which are homologues of Gtr1/2 [49]. As described, mTORC1 binds to the Ulk1 complex; therefore, the mTORC1-Ulk1 complex may also reside on lysosomal membrane during nutrient rich condition. The detailed mechanism for how such localization shifts affect autophagy regulation is still to be determined.

## 5. Gene Expression 

In yeast, another representative contributor to TORC1 regulation of autophagy is Atg8. Atg8 is a ubiquitin-like protein linked to a lipid molecule, phosphatidylethanolamine (PE), instead of proteins, and thus is different from the other ubiquitin-related proteins [50]. In response to nitrogen starvation or TORC1 inactivation, the expression of Atg8 mRNA is robustly increased [51,52]. By artificially lowering the expression, the size of autophagosomes is decreased [53]. The actual function of Atg8 in autophagosome formation is still under discussion, but it is possible that the two different membrane vesicles on which Atg8 is anchored via PE create hemi-fusion [54]. The recruitment of Atg8 to the autophagosome formation site is the last step in the hierarchy of all Atg proteins [21]. Therefore, it is assumed that Atg8 serves to elongate the autophagosomal membrane, at least in yeast. Rph1, a histone demethylase protein, serves as a repressor of Atg8 mRNA under nutrient-rich conditions, together with its homologue, Gis1 [55]. Phosphorylation of Rph1 is responsible for its suppression function, and is catalyzed by Rim15 protein kinase, which is under regulation of TORC1 at least partly [55]. This Rph1 dependent system regulates Atg1, 7, 9, 14 and 29 expression either [55]. The chromatin remodeler Rsc1-RSC complex is required for proper Atg8 expression, but its relation to TORC1 is not known [56]. In addition, Atg8 expression is negatively regulated by the Ume6-Sin3-Rpd3 histone deacetylase complex under growth conditions [57]. This Ume6-Sin3-Rpd3 dependent regulation seems to be under control of Rim15, which is under control of the TORC1, Sch9 and cAMP dependent kinase/protein kinase A (PKA), and cyclin-dependent kinase Pho85 [57,58]. The relationship between TORC1 and PKA pathways seems probable, but a detailed connection is not yet determined. It would be interesting to investigate whether TORC1 directly affects Atg8 expression in addition to affecting the PKA axis. An interesting protein in this regard is the Ksp1 protein kinase [59]. In the absence of Ksp1, autophagy is hyper-induced. It is phosphorylated by PKA and alters TORC1-dependent Atg13 phosphorylation. Thus, it is proposed that Ksp1 connects the PKA and TORC1 axis. 

TORC1 is also involved in the mRNA degradation of Atg genes represented by Atg8, through decapping by RNA helicase RCK family members in association with the decapping enzyme Dcp2 both in yeast and mammals [60]. Dcp2 is proposed to be phosphorylated by TORC1 under nutrient-rich conditions, which facilitates degradation of Atg gene mRNA [60]. 

TORC1 is also involved in starvation-induced autophagy through regulation of vacuolar protease expression in yeast. The expression of representative vacuolar proteases, such as protease B and protease A, is induced in response to nitrogen starvation. This regulation is catalyzed by GATA-family transcription factors Gln3, Gat1, and Dal80 [61]. Localization of Gln3 to the nucleus, and its subsequent activity, is regulated by the phosphorylation status of Gln3 and its binding partner Ure2 [62]. When TORC1 is inactivated, the downstream phosphatases are activated, and Gln3 is dephosphorylated [63]. Gln3 is also important for upregulation of Atg14 expression during starvation [64]. In addition to this well-established model, recent studies proposed that GCN2/GCN4 dependent general amino acid control (GAAC) pathway is responsible for Gln3 localization regulation [65]. This uncharged tRNA-mediated amino acid responsive pathway may be interconnected with TORC1-dependent pathways. Based on recent observations that uncharged tRNA have a negative impact on TORC1 activity, uncharged tRNA-mediated processes may have some impact on TORC1 regulatory mechanisms [66]. Mammalian cells have similarly sophisticated mTORC1-mediated systems regulation of lysosomal gene expression regulation. Transcriptional factor EB (TFEB) is phosphorylated by active mTORC1 under nutrient-rich conditions and retained in the cytosol [67,68]. After starvation, dephosphorylated TFEB enters the nucleus and globally upregulates lysosome-related genes such as cathepsin and V-ATPase as well as autophagy-related genes such as Atg9B [67,68]. All these regulatory mechanisms could facilitate the catabolic process of autophagy.

## 6. Future Directions

Thus, in the last two decades, we have developed a better understanding of the regulation of protein degradation. One less-characterized connection between TORC1 and autophagy may be found in PI3P metabolism. It is known that a PIP3 kinase (Vps34) complex specific to autophagosome formation complex exists in both yeast and mammals [69,70,71]. One of the mammalian subunits of this complex, Beclin-1, is reported to be phosphorylated by Ulk1 in response to mTORC1 suppression under starvation conditions [72]. As a result, formation of the Vps34 complex is enhanced and autophagy is induced. Although it is known that local concentration of PI3P at the site of autophagosome formation needs to be properly controlled, the precise mechanism underlying the regulation of PI3P kinase remains to be investigated. Overexpression of an inactive form of PI3-phosphatase, MTMR3, led to increased local PI3P concentration at the autophagosome formation site, which initiated autophagy without starvation stimulation [73]. However, this study left a question as to why an mTORC1-mediated step could be bypassed. Recently, it was found that MTMR3 directly binds to mTORC1 and suppresses its activity [74]. This could explain why autophagy was induced without starvation stimulation. As proposed in the past, there may be some connection between mTORC1 regulation and PI3P metabolism [75]. 

Furthermore it is clear that degradation products of autophagy, such as amino acids, activate TORC1 [76], suggesting a negative feedback loop regulation. Where are such amino acid sensed in cell? Inside the lysosome? Or in the cytosol? When and how is autophagy terminated? Is TORC1 involved in such regulation? Several questions remain to be addressed and may need decades before we have a more comprehensive picture of autophagy regulation.

## References

[1] Schoenheimer R. (1942). The Dynamic State of Body Constituents.

[2] Ashoford T.P., Porter K.R. (1962). Cytoplasmic components in hepatic cell lysosomes. J. Cell Biol..

[3] Tsukada M., Ohsumi Y. (1993). Isolation and characterization of autophagy-defective mutants of *Saccharomyces cerevisiae*. FEBS Lett..

[4] Thumm M., Egner R., Koch B., Schlumpberger M., STRAUB M., Veenhuis M., Wolf D.H. (1994). Isolation of Autophagocytosis Mutants of *Saccharomyces cerevisiae*. FEBS Lett..

[5] Harding T.M., Morano K.A., Scott S.V., Klionsky D.J. (1995). Isolation and characterization of yeast mutants in the cytoplasm to vacuole protein targeting pathway. J. Cell Biol..

[6] Blommaart E., Luiken J., Blommaart P., Vanwoerkom G.M., Meijer A.J. (1995). Phosphorylation of Ribosomal-Protein S6 Is Inhibitory for Autophagy in Isolated Rat Hepatocytes. J. Biol. Chem..

[7] Noda T., Ohsumi Y. (1998). Tor, a phosphatidylinositol kinase homologue, controls autophagy in yeast. J. Biol. Chem..

[8] Guo J.Y., White E. (2016). Autophagy, Metabolism, and Cancer. Cold Spring Harb. Symp. Quant. Biol..

[9] Funakoshi T., Matsuura A., Noda T., Ohsumi Y. (1997). Analyses of APG13 gene involved in autophagy in yeast, *Saccharomyces cerevisiae*. Gene.

[10] Kamada Y., Funakoshi T., Shintani T., Nagano K., Ohsumi M., Ohsumi Y. (2000). Tor-mediated induction of autophagy via an Apg1 protein kinase complex. J. Cell Biol..

[11] Kamada Y., Yoshino K.-I., Kondo C., Kawamata T., Oshiro N., Yonezawa K., Ohsumi Y. (2010). Tor directly controls the Atg1 kinase complex to regulate autophagy. Mol. Cell. Biol..

[12] Yeasmin A.M., Waliullah T.M., Kondo A., Kaneko A., Koike N., Ushimaru T. (2016). Orchestrated Action of PP2A Antagonizes Atg13 Phosphorylation and Promotes Autophagy after the Inactivation of TORC1. PLoS ONE.

[13] Stephan J.S., Yeh Y.-Y.Y., Ramachandran V., Deminoff S.J., Herman P.K. (2009). The Tor and PKA signaling pathways independently target the Atg1/Atg13 protein kinase complex to control autophagy. Proc. Natl. Acad. Sci. USA.

[14] Fujioka Y., Suzuki S.W., Yamamoto H., Kondo-Kakuta C., Kimura Y., Hirano H., Akada R., Inagaki F., Ohsumi Y., Noda N.N. (2014). Structural basis of starvation-induced assembly of the autophagy initiation complex. Nat. Struct. Mol. Biol..

[15] Yamamoto H., Fujioka Y., Suzuki S.W., Noshiro D., Suzuki H., Kondo-Kakuta C., Kimura Y., Hirano H., Ando T., Noda N.N. (2016). The Intrinsically Disordered Protein Atg13 Mediates Supramolecular Assembly of Autophagy Initiation Complexes. Dev. Cell.

[16] Kabeya Y., Noda N.N., Fujioka Y., Suzuki K., Inagaki F., Ohsumi Y. (2009). Characterization of the Atg17–Atg29–Atg31 complex specifically required for starvation-induced autophagy in *Saccharomyces cerevisiae*. Biochem. Biophys. Res. Commun..

[17] Ragusa M.J., Stanley R.E., Hurley J.H. (2012). Architecture of the Atg17 Complex as a Scaffold for Autophagosome Biogenesis. Cell.

[18] Matsuura A., Tsukada M., Wada Y., Ohsumi Y. (1997). Apg1p, a novel protein kinase required for the autophagic process in *Saccharomyces cerevisiae*. Gene.

[19] Yeh Y.-Y.Y., Shah K.H., Herman P.K. (2011). An Atg13 protein-mediated self-association of the Atg1 protein kinase is important for the induction of autophagy. J. Biol. Chem..

[20] Suzuki K., Kirisako T., Kamada Y., Mizushima N., Noda T., Ohsumi Y. (2001). The pre-autophagosomal structure organized by concerted functions of APG genes is essential for autophagosome formation. EMBO J..

[21] Suzuki K., Kubota Y., Sekito T., Ohsumi Y. (2007). Hierarchy of Atg proteins in pre-autophagosomal structure organization. Genes Cells.

[22] Ueno T., Ishidoh K., Mineki R., Tanida I., Murayama K., Kadowaki M., Kominami E. (1999). Autolysosomal membrane-associated betaine homocysteine methyltransferase. Limited degradation fragment of a sequestered cytosolic enzyme monitoring autophagy. J. Biol. Chem..

[23] Yan J., Kuroyanagi H., Kuroiwa A., Matsuda Y., Tokumitsu H., Tomoda T., Shirasawa T., Muramatsu M. (1998). Identification of mouse ULK1, a novel protein kinase structurally related to *C. elegans* UNC-51. Biochem. Biophys. Res. Commun..

[24] Hosokawa N., Hara T., Kaizuka T., Kishi C., Takamura A., Miura Y., Iemura S.-I., Natsume T., Takehana K., Yamada N. (2009). Nutrient-dependent mTORC1 association with the ULK1–Atg13–FIP200 complex required for autophagy. Mol. Biol. Cell.

[25] Jung C.H., Jun C.B., Ro S.-H., Kim Y.-M., Otto N.M., Cao J., Kundu M., Kim D.-H. (2009). ULK–Atg13–FIP200 complexes mediate mTOR signaling to the autophagy machinery. Mol. Biol. Cell.

[26] Chan E.Y.W., Longatti A., McKnight N.C., Tooze S.A. (2009). Kinase-inactivated ULK proteins inhibit autophagy via their conserved C-terminal domains using an Atg13-independent mechanism. Mol. Cell. Biol..

[27] Hara T., Takamura A., Kishi C., Iemura S.-I., Natsume T., Guan J.-L., Mizushima N. (2008). FIP200, a ULK-interacting protein, is required for autophagosome formation in mammalian cells. J. Cell Biol..

[28] Mercer C.A., Kaliappan A., Dennis P.B. (2009). A novel, human Atg13 binding protein, Atg101, interacts with ULK1 and is essential for macroautophagy. Autophagy.

[29] Hosokawa N., Sasaki T., Iemura S.-I., Natsume T., Hara T., Mizushima N. (2009). Atg101, a novel mammalian autophagy protein interacting with Atg13. Autophagy.

[30] Itakura E., Mizushima N. (2010). Characterization of autophagosome formation site by a hierarchical analysis of mammalian Atg proteins. Autophagy.

[31] Papinski D., Schuschnig M., Reiter W., Wilhelm L., Barnes C.A., Maiolica A., Hansmann I., Pfaffenwimmer T., Kijanska M., Stoffel I. (2014). Early Steps in Autophagy Depend on Direct Phosphorylation of Atg9 by the Atg1 Kinase. Mol. Cell.

[32] Noda T., Kim J., Huang W.-P., Baba M., Tokunaga C., Ohsumi Y., Klionsky D.J. (2000). Apg9p/Cvt7p is an integral membrane protein required for transport vesicle formation in the Cvt and autophagy pathways. J. Cell Biol..

[33] Reggiori F., Tucker K.A., Stromhaug P.E., Klionsky D.J. (2004). The Atg1-Atg13 complex regulates Atg9 and Atg23 retrieval transport from the pre-autophagosomal structure. Dev. Cell.

[34] Shirahama-Noda K., Kira S., Yoshimori T., Noda T. (2013). TRAPPIII is responsible for vesicular transport from early endosomes to Golgi, facilitating Atg9 cycling in autophagy. J. Cell Sci..

[35] Yamamoto H., Kakuta S., Watanabe T.M., Kitamura A., Sekito T., Kondo-Kakuta C., Ichikawa R., Kinjo M., Ohsumi Y. (2012). Atg9 vesicles are an important membrane source during early steps of autophagosome formation. J. Cell Biol..

[36] Suzuki S.W., Yamamoto H., Oikawa Y., Kondo-Kakuta C., Kimura Y., Hirano H., Ohsumi Y. (2015). Atg13 HORMA domain recruits Atg9 vesicles during autophagosome formation. Proc. Natl. Acad. Sci. USA.

[37] Rao Y., Perna M.G., Hofmann B., Beier V., Wollert T. (2016). The Atg1-kinase complex tethers Atg9-vesicles to initiate autophagy. Nat. Commun..

[38] Karanasios E., Walker S.A., Okkenhaug H., Manifava M., Hummel E., Zimmermann H., Ahmed Q., Domart M.-C., Collinson L., Ktistakis N.T. (2016). Autophagy initiation by ULK complex assembly on ER tubulovesicular regions marked by ATG9 vesicles. Nat. Commun..

[39] Mizushima N., Yoshimori T., Ohsumi Y. (2011). The Role of Atg Proteins in Autophagosome Formation. Annu. Rev. Cell Dev. Biol..

[40] Yin Z., Pascual C., Klionsky D.J. (2016). Autophagy: Machinery and regulation. Microb. Cell.

[41] Sturgill T.W., Cohen A., Diefenbacher M., Trautwein M., Martin D.E., Hall M.N. (2008). TOR1 and TOR2 have distinct locations in live cells. Eukaryotic Cell.

[42] Dubouloz F., Deloche O., Wanke V., Cameroni E., de Virgilio C. (2005). The TOR and EGO protein complexes orchestrate microautophagy in yeast. Mol. Cell.

[43] Kira S., Kumano Y., Ukai H., Takeda E., Matsuura A., Noda T. (2015). Dynamic relocation of the TORC1–Gtr1/2–Ego1/2/3 complex is regulated by Gtr1 and Gtr2. Mol. Biol. Cell.

[44] Powis K., Zhang T., Panchaud N., Wang R., de Virgilio C., Ding J. (2015). Crystal structure of the Ego1–Ego2–Ego3 complex and its role in promoting Rag GTPase-dependent TORC1 signaling. Cell Res..

[45] Jeong J.-H., Lee K.-H., Kim Y.-M., Kim D.-H., Oh B.-H., Kim Y.-G. (2012). Crystal Structure of the Gtr1p^GTP^-Gtr2p^GDP^ Protein Complex Reveals Large Structural Rearrangements Triggered by GTP-to-GDP Conversion. J. Biol. Chem..

[46] Binda M., Péli-Gulli M.-P., Bonfils G., Panchaud N., Urban J., Sturgill T.W., Loewith R., de Virgilio C. (2009). The Vam6 GEF controls TORC1 by activating the EGO complex. Mol. Cell.

[47] Kira S., Tabata K., ShirahamaNoda K., Nozoe A., Yoshimori T., Noda T. (2014). Reciprocal conversion of Gtr1 and Gtr2 nucleotide-binding states by Npr2–Npr3 inactivates TORC1 and induces autophagy. Autophagy.

[48] Ukai H., Araki Y., Kira S., Noda T. (2017). Osaka University, Osaka, Japan.

[49] Bar-Peled L., Sabatini D.M. (2014). Regulation of mTORC1 by amino acids. Trends Cell Biol..

[50] Ichimura Y., Kirisako T., Takao T., Satomi Y., Shimonishi Y., Ishihara N., Mizushima N., Tanida I., Kominami E., Ohsumi M. (2000). A ubiquitin-like system mediates protein lipidation. Nature.

[51] Kirisako T., Baba M., Ishihara N., Miyazawa K., Ohsumi M., Yoshimori T., Noda T., Ohsumi Y. (1999). Formation process of autophagosome is traced with Apg8/Aut7p in yeast. J. Cell Biol..

[52] Huang W.-P., Scott S.V., Kim J., Klionsky D.J. (2000). The itinerary of a vesicle component, Aut7p/Cvt5p, terminates in the yeast vacuole via the autophagy/Cvt pathways. J. Biol. Chem..

[53] Abeliovich H., Dunn W.A.J., Kim J., Klionsky D.J. (2000). Dissection of autophagosome biogenesis into distinct nucleation and expansion steps. J. Cell Biol..

[54] Nakatogawa H., Ichimura Y., Ohsumi Y. (2007). Atg8, a Ubiquitin-like Protein Required for Autophagosome Formation, Mediates Membrane Tethering and Hemifusion. Cell.

[55] Bernard A., Jin M., González-Rodríguez P., Füllgrabe J., Delorme-Axford E., Backues S.K., Joseph B., Klionsky D.J. (2015). Rph1/KDM4 Mediates Nutrient-Limitation Signaling that Leads to the Transcriptional Induction of Autophagy. Curr. Biol..

[56] Yu F., Imamura Y., Ueno M., Suzuki S.W., Ohsumi Y., Yukawa M., Tsuchiya E. (2015). The yeast chromatin remodeler Rsc1–RSC complex is required for transcriptional activation of autophagy-related genes and inhibition of the TORC1 pathway in response to nitrogen starvation. Biochem. Biophys. Res. Commun..

[57] Bartholomew C.R., Suzuki T., Du Z., Backues S.K., Jin M., Lynch-Day M.A., Umekawa M., Kamath A., Zhao M., Xie Z. (2012). Ume6 transcription factor is part of a signaling cascade that regulates autophagy. Proc. Natl. Acad. Sci. USA.

[58] Yang Z., Geng J., Yen W.-L., Wang K., Klionsky D.J. (2010). Positive or negative roles of different cyclin-dependent kinase Pho85–cyclin complexes orchestrate induction of autophagy in *Saccharomyces cerevisiae*. Mol. Cell.

[59] Umekawa M., Klionsky D.J. (2012). Ksp1 kinase regulates autophagy via the target of rapamycin complex 1 (TORC1) pathway. J. Biol. Chem..

[60] Hu G., McQuiston T., Bernard A., Park Y.-D., Qiu J., Vural A., Zhang N., Waterman S.R., Blewett N.H., Myers T.G. (2015). A conserved mechanism of TOR-dependent RCK-mediated mRNA degradation regulates autophagy. Nat. Cell Biol..

[61] Coffman J.A., Cooper T.G. (1997). Nitrogen GATA factors participate in transcriptional regulation of vacuolar protease genes in *Saccharomyces cerevisiae*. J. Bacteriol..

[62] Kulkarni A.A., Abul-Hamd A.T., Rai R., El Berry H., Cooper T.G. (2001). Gln3p nuclear localization and interaction with Ure2p in *Saccharomyces cerevisiae*. J. Biol. Chem..

[63] Cooper T.G. (2002). Transmitting the signal of excess nitrogen in *Saccharomyces cerevisiae* from the Tor proteins to the GATA factors: Connecting the dots. FEMS Microbiol. Rev..

[64] Chan T.F., Bertram P.G., Ai W., Zheng X.F. (2001). Regulation of APG14 expression by the GATA-type transcription factor Gln3p. J. Biol. Chem..

[65] Tate J.J., Buford D., Rai R., Cooper T.G. (2017). General Amino Acid Control and 14-3-3 Proteins Bmh1/2 Are Required for Nitrogen Catabolite Repression-Sensitive Regulation of Gln3 and Gat1 Localization. Genetics.

[66] Kamada Y. (2017). Novel tRNA function in amino acid sensing of yeast Tor complex1. Genes Cells.

[67] Sardiello M., Palmieri M., di Ronza A., Medina D.L., Valenza M., Gennarino V.A., Di Malta C., Donaudy F., Embrione V., Polishchuk R.S. (2009). A Gene Network Regulating Lysosomal Biogenesis and Function. Science.

[68] Settembre C., Di Malta C., Polito V.A., Garcia Arencibia M., Vetrini F., Erdin S., Erdin S.U., Huynh T., Medina D., Colella P. (2011). TFEB links autophagy to lysosomal biogenesis. Science.

[69] Kihara A., Noda T., Ishihara N., Ohsumi Y. (2001). Two distinct Vps34 phosphatidylinositol 3-kinase complexes function in autophagy and carboxypeptidase Y sorting in *Saccharomyces cerevisiae*. J. Cell Biol..

[70] Itakura E., Kishi C., Inoue K., Mizushima N. (2008). Beclin 1 forms two distinct phosphatidylinositol 3-kinase complexes with mammalian Atg14 and UVRAG. Mol. Biol. Cell.

[71] Matsunaga K., Saitoh T., Tabata K., Omori H., Satoh T., Kurotori N., Maejima I., ShirahamaNoda K., Ichimura T., Isobe T. (2009). Two Beclin 1-binding proteins, Atg14L and Rubicon, reciprocally regulate autophagy at different stages. Nat. Cell Biol..

[72] Russell R.C., Tian Y., Yuan H., Park H.W., Chang Y.-Y., Kim J., Kim H., Neufeld T.P., Dillin A., Guan K.-L. (2013). ULK1 induces autophagy by phosphorylating Beclin-1 and activating VPS34 lipid kinase. Nat. Cell Biol..

[73] Taguchi-Atarashi N., Hamasaki M., Matsunaga K., Omori H., Ktistakis N.T., Yoshimori T., Noda T. (2010). Modulation of local PtdIns3P levels by the PI phosphatase MTMR3 regulates constitutive autophagy. Traffic.

[74] Hao F., Itoh T., Morita E., ShirahamaNoda K., Yoshimori T., Noda T. (2016). The PtdIns3-phosphatase MTMR3 interacts with mTORC1 and suppresses its activity. FEBS Lett..

[75] Nobukuni T., Kozma S.C., Thomas G. (2007). hvps34, an ancient player, enters a growing game: mTOR Complex1/S6K1 signaling. Curr. Opin. Cell Biol..

[76] Shigemitsu K., Tsujishita Y., Hara K., Nanahoshi M., Avruch J., Yonezawa K. (1999). Regulation of translational effectors by amino acid and mammalian target of rapamycin signaling pathways. Possible involvement of autophagy in cultured hepatoma cells. J. Biol. Chem..

